# Supplementary material for: Complexome profiling of the Chlamydomonas psb28 mutant reveals TEF5 as an early PSII assembly factor
Source: Plant Cell. 2025 Mar 17;37(6):koaf055. doi: 10.1093/plcell/koaf055 (PMC12164590; doi:10.1093/plcell/koaf055)
Supplement: koaf055_Supplementary_Data [file koaf055_supplementary_data.zip › psb28_interactive.zip!/plots/intensity/qualityPlots/peptides/HSLV1_Cre14.g628350.t1.2.html]

### Cre14.g628350.t1.2

Trivial name: HSLV1  
SeqCoverage [median]: 25.00  
Mass: 25.61 kDa  
  
 

| Sequence | MissedCleavage | Mass [Da] | wtIntensity | muIntensity | Score |
| --- | --- | --- | --- | --- | --- |
| AAVELAK | 0 | 700.41 | 1896.82 | 5197.79 | 128.82 |
| ALMDVPEMDAMTIGTK | 0 | 1721.80 | 813.76 | 0.00 | 72.34 |
| KDDMVVMIGDGQVSVGSVSVKPNVR | 2 | 2615.34 | 942.16 | 3398.38 | 70.53 |
| LEEHPGQLLR | 0 | 1190.64 | 660.73 | 1112.09 | 77.66 |
| LEMKLEEHPGQLLR | 1 | 1691.90 | 0.00 | 2417.20 | 59.25 |
|  |  |  | 4313.5 | 12125.5 |  |
